# ICFSR Task Force Perspective on Biomarkers for Sarcopenia and Frailty

**DOI:** 10.14283/jfa.2019.32

**Published:** 2019-10-07

**Authors:** L. Rodriguez-Mañas, I. Araujo de Carvalho, S. Bhasin, H.A. Bischoff-Ferrari, M. Cesari, W. Evans, J.M. Hare, M. Pahor, A. Parini, Y. Rolland, R.A. Fielding, J. Walston, B. Vellas, The ICFSR Task Force

**Affiliations:** 1 Servicio de Geriatría, Hospital Universitario de Getafe, Toledo, Spain; 2 World Health Organization, Geneva, Switzerland; 3 Brigham and Women's Hospital, Harvard Medical School, Boston, MA, USA; 4 University Hospital and University of Zurich, Zurich, Switzerland; 5 Fondazione IRCCS Ca' Granda Ospedale Maggiore Policlinico, University of Milan, Milan, Italy; 6 Duke University Medical Center, Durham, NC, USA; 7 Interdisciplinary Stem Cell Institute, University of Miami Miller School of Medicine, Miami, FL, USA; 8 University of Florida Institute on Aging, Gainesville, FL, USA; 9 Institute of Cardiovascular and Metabolic Diseases, INSERM U1048, CHU Toulouse, Toulouse, France; 10 Service de Médecine Interne et Gérontologie, Clinique Gérontopôle, Hôpital La Crave, Casselardit, Toulouse, France; 11 Tufts University, Boston, MA, USA; 12 Johns Hopkins Division of Geriatric Medicine and Gerontology, Baltimore, Maryland, USA; 13 Gerontopole, INSERM U1027, Alzheimer's Disease Research and Clinical Center, Toulouse University Hospital, Toulouse, France

**Keywords:** Frailty, sarcopenia, biomarkers, consensus

## Abstract

Biomarkers of frailty and sarcopenia are essential to advance the understanding of these conditions of aging and develop new diagnostic tools and effective treatments. The International Conference on Frailty and Sarcopenia Research (ICFSR) Task Force - a group of academic and industry scientists from around the world — met in February 2019 to discuss the current state of biomarker development for frailty and sarcopenia. The D3Cr dilution method, which assesses creatinine excretion as a biochemical measure of muscle mass, was suggested as a more accurate measure of functional muscle mass than assessment by dual energy x-ray absorptiometry (DXA). Proposed biomarkers of frailty include markers of inflammation, the hypothalamic-pituitary-adrenal (HPA) axis response to stress, altered glucose insulin dynamics, endocrine dysregulation, aging, and others, acknowledging the complex multisystem etiology that contributes to frailty. Lack of clarity regarding a regulatory pathway for biomarker development has hindered progress; however, there are currently several international efforts to develop such biomarkers as tools to improve the treatment of individuals presenting these conditions.

## Introduction

Biomarkers have proven essential to advance understanding of the biological underpinnings of various diseases, as diagnostic tools, and in clinical trials as indicators of treatment effectiveness. For complex conditions such as sarcopenia and frailty, the multiplicity of phenotypes and pathogenic mechanisms makes the development of biomarkers particularly challenging, since biological markers associated with single aspects of the condition are only marginally associated with clinically relevant outcomes (1).

In February 2019, the International Conference on Frailty and Sarcopenia Research (ICSFR) Task Force convened a meeting to discuss the current status of biomarker development for sarcopenia and frailty. The ICFSR Task Force comprises academic and industry scientists from 13 countries in North America, Europe, Asia, and Australia/Oceania who are involved in the development of interventions to treat these disabling age-related conditions.

The term sarcopenia was coined by Rosenberg in the late' 80s to describe age-related loss of muscle mass and was later revised to incorporate declines in muscle strength and physical function (2, 3). Assessment of muscle mass by dual energy x-ray absorptiometry (DXA), computed tomography (CT), and magnetic resonance imaging (MRI) have provided the most widely used biomarkers for sarcopenia (4). However, CT and MRI have limitations related to the high cost and complexity of the technology, and DXA has shown poor correlation with health-related quality of life (5).

Frailty is a syndrome characterized by progressive functional decline, decreased physiological reserve and resilience and increased vulnerability to a variety of stressors (6). Multiple operational definitions of frailty have been proposed (7, 8, 9). The phenotypic criteria proposed by Fried and colleagues, which define frailty by the presence of weakness, slowness, weight loss, declining physical function, and fatigue continue to be the most widely used (10).

For both frailty and sarcopenia, identification of biomarkers depends on the definition of the condition and the goal is to develop clinically relevant markers as diagnostic tools, to assess treatment effectiveness, to understand biological etiology, and to advance prevention efforts. In 2016, an International Classification of Diseases, Tenth Revision, Clinical Modification (ICD-10-CM) code was established for sarcopenia, which enabled it to be recognized by the US Food and Drug Administration (FDA) and European Medicines Agency (EMA) and the National Centre for Classification in Health (NCCH) in Australia as a separately reportable condition (11). By removing barriers for diagnosing sarcopenia, the ICD-10 code will enable standardized data collection and improve the efficiency of clinical trials (12). However, the presence of multiple, largely overlapping operational definitions and the multidimensional nature of frailty make it unlikely that an ICD code for frailty will be established in the near future.

Frailty is strongly associated with muscle mass and function; thus, sarcopenia has been proposed as the biological substrate for physical frailty (13). Merging the two conditions into a single entity - Physical Frailty and Sarcopenia (PF&S) - a condition that can be diagnosed and potentially treated has also been proposed (14, 15) and a core inflammatory profile with a gender-specific signature has been identified (16).

## Biomarkers of sarcopenia and frailty

A definition of sarcopenia should take into account the role of muscle mass in the risk of disability and age-related risk of chronic disease; thus, for sarcopenia measures of muscle mass, quality, and function have been proposed as potential biomarkers. Plasma growth and differentiation factor-15 (GDF-15) has also been associated with sarcopenia-related outcomes and increases with age but has not been evaluated as a sarcopenia biomarker (17). Since frailty has a complex multisystem etiology, biomarkers needed to assess multiple dysregulated systems. Proposed biomarkers of frailty include inflammatory markers such as interleukin-6 (IL-6), tumor necrosis factor-alpha (TNF-α), C-reactive protein (CRP), neutrophil cell count (18), and others (Table 1).

**Table 1:** Possible Biomarkers of Frailty and Sarcopenia

Condition	Attribute	Method	References
Sarcopenia	Muscle Mass	D_3_Cr dilution DXA total lean mass DXA ALM/ht^2^	22–23
	Muscle atrophy	GDF-15	17
	Muscle function		
	Muscle quality		
Frailty	Inflammation Chronic inflammation	IL-6, TNF-α, CRP, Neutrophil cell count Inflammation index score	18
	Stress response	Salivary cortisol	28,29
	Altered glucose insulin dynamics	Serum insulin-like growth factor,	30, 34
	Endocrine dysregulation	Testosterone, estradiol,	31, 32, 33
	Endothelial dysfunction		36
	Clotting factors		37
	Mitochondrial dysfunction		38
	Alterations in metabolome		29
	Markers of aging		40–44
	Vaccine responsiveness		
	Other	SIRT1	33
Physical Frailty and Sarcopenia (PF&S)	Core inflammatory profile	P-selectin, CRP, IFNγ-induced protein 10, MPO, IL-8, MCP-1, macrophage inflammatory protein 1-α, PDGF-BB,	16


CRP, C-reactive protein; DXA, dual X-ray absorptiometry; GDF-15, growth differentiation factor 15; IFNγ, interferon gamma; IL-6, interleukin 6; IL-8, interleukin 8; MCP-1, monocyte chemoattractant protein 1; MPO, myeloperoxidase; PDGF-BB, platelet derived growth factor BB; SIRT, silent mating-type information regulation 2 homolog 1; TNF-α, tumor necrosis factor alpha.

### D3-Creatine (D3Cr) dilution — a biomarker for sarcopenia

Although dozens of papers measure lean mass and call it muscle mass, lean mass is not the same as muscle mass. Indeed, the Foundation of the National Institutes of Health (FNIH) Sarcopenia project concluded that low lean mass is poor predictor of functional impairment (19). The relationship between muscle mass measured by XX and fracture risk is highly significant; however appendicular lean mass (ALM) is not related to what? at all (20).

To assess the true effects of intrinsic, age-associated effects on skeletal muscle contractile function, an accurate measure of functional muscle mass undiluted by lipid, connective tissue, and fibrotic tissue is needed. Assessment of creatinine excretion provides such a measure of muscle mass (21). Creatine is irreversibly converted to creatinine and excreted in urine, where it can be measured by liquid chromatography-mass spectrometry (LCMS). Evans and colleagues developed a direct and accurate method for measuring creatine pool size by orally administering stable isotope-labelled creatine and then collecting a single fasted urine sample 48–96 hours after dosing for measurement of D_3_Cr (22, 23). This D_3_Cr dilution method uses an algorithm based on urine levels of creatine and creatinine to determine the dilution of the oral label in the whole-body creatine pool of skeletal muscle, thus providing an accurate measure of skeletal muscle mass.

In the Osteoporotic Fractures in Men (MrOS) study, a multisite study of community dwelling men 80 years and older, the D_3_Cr dilution method was compared to DXA, high-resolution peripheral quantitative CT (HRpQCT), Short Physical Performance Batter (SPPB), the 400-meter walk test (400MW), and force plate for lower extremity power. Muscle mass by the D_3_Cr dilution method showed a moderate correlation with DXA total lean mass but no correlation with DXA ALM/ht^2^. It also demonstrated a strong relation between muscle mass determined by D_3_Cr dilution method with physical performance (SPPB, chair stands), incidence of falls, and mobility limitations (20). In assessing the relative importance of muscle versus fat in sarcopenic obesity, repeated assessment of multiple measures at 18-month intervals showed that muscle mass determined using the D_3_Cr dilution method correlated with grip strength and walking speed even though there was no change in total lean mass, ALM, or ALM/ht^2^. Muscle mass determined using the D_3_Cr dilution method also was shown to be a strong predictor of disability (24). These results suggest that muscle mass is a primary determinant of physical performance and adverse outcomes, and that the relative effects of higher body fatness are less important. However, results regarding the D3Cr dilution method need to be replicated in large representative cohorts.

### Frailty biomarkers

Potential biological triggers of frailty in older adults may include increased inflammation and mitophagy (25); altered stress response systems mediated through the angiotensin system, the HPA axis, and the sympathetic nervous system; and decreased energy production. Chronic inflammatory markers such as IL-6, CRP, interleukin-1-receptor agonist, interleukin-18, and soluble TNF-a receptor 1 (sTNFR1), combined in an inflammation index score, appears to capture the magnitude of chronic inflammation in aging and was shown to be a better predictor of mortality compared to single measures (26). However, these markers are highly variable and non-specific and influenced by meals and time of day. Recent studies suggest that sTNFR1 is the least variable over weeks and months.

Salivary cortisol has been used as a marker of the hypothalamic-pituitary-adrenal (HPA) axis response to stress (27), and a diurnal pattern of cortisol levels (lower in the morning, higher in the evening) has been associated with frailty (28, 29). Frailty has also been associated with lower levels of serum insulin-like growth factor (30), lower levels of testosterone and high levels of estradiol (31, 32), elevated levels of silent mating-type information regulation 2 homolog 1 (SIRT1) (33), altered glucose-insulin dynamics (34), endocrine dysregulation (35), endothelial dysfunction (36), elevated clotting factors (37), mitochondrial dysfunction (38), and alterations in the metabolome (39).

Given that frailty is an aging-related syndrome, biomarkers of aging are also important and have been gaining increased attention with the emergence of the field of gerosciences (40, 41, 42). For example, possible biomarkers of frailty include a marker of nuclear membrane defects, which has been associated with aging (43), the expression of several mRNAs involved in the cell response to stress (44), and markers of mTOR activation, the adaptive immune system, and cell senescence.

Age-related changes in the adaptive and innate immune response including the chronic low-level proinflammatory state known as inflammaging, and immunosenescence, which is strongly driven by inflammaging, result in increased susceptibility to influenza and other disease and a decreased response to influenza vaccination (6, 45, 46, 47). Hare and colleagues have been developing mesenchymal stem cells (MSCs) as a treatment for many diseases of aging, including frailty. They have shown that MSCs improve immune potential by modulating T and B cell response. Moreover, their studies suggest that vaccine responsiveness may represent an ideal biomarker of aging in that it correlates with the frailty phenotype, changes with interventions that change the phenotype (such as MSC treatment), represents a biologically plausible mechanism of frailty, and provides medically meaningful information.

## Regulatory considerations

While frailty is an acceptable concept in clinical care and for characterizing populations, it is not presently a “disease entity” recognized by and ICD-10 code. However, to adapt our health care system to an aging population, transitioning from a disease-centered to a function-centered approach will be necessary to maintain function in older adults and prevent dependency. For these reasons, biomarkers of frailty are urgently needed. Frailty is an entity where several physiological systems are dysregulated or malfunctioning. Thus, an isolated biomarker of frailty would have limited usefulness in drug development whereas the search for panels of biomarkers seems promising.

Context of use is an important consideration for regulators. Biomarkers are useful as indicators of target engagement or for screening, diagnosis, or assessing outcomes in specifically-designated populations. Consensus from the field on what would represent an appropriate biomarker/set of biomarkers for proof of concept versus clinical trials could support efforts to achieve regulatory acceptance. However, it is necessary that the physiopathological mechanisms underlying the two conditions of interest are carefully defined and limited in order to propose unequivocal biomarkers of “disease”. Ongoing projects such as the Sarcopenia and Physical fRailty IN older people: multi-componenT Treatment strategies (SPRINTT), funded by the Innovative Medicines Initiative (IMI), are going in exactly this direction (48). Running in parallel with SPRINTT, the BIOmarkers associated with Sarcopenia and Physical frailty in EldeRly pErsons (BIOSPHERE) study analyzed 12 candidate serum biomarkers to identify and validate a panel of PF&S biomarkers that capture the multi-factorial nature of PF&S, identify potential intervention targets, and provide potential diagnostic tools and endpoints for use in clinical trials (49). In this same regard, FRAILOMICS is evaluating the role of sets of biomarkers in the prediction of the risk of developing physical frailty, its diagnosis and its prognosis in terms of incident disability and death (50, 51).

## Conclusions

Recognizing that the field is in the early stages of developing biomarkers for sarcopenia and frailty, the Task Force identified several research gaps and barriers that need to be addressed to expedite this process and move biomarkers from research to clinical settings.

Part of the difficulty resides in the difficulty of applying the usual standards applicable to stand-alone diseases of young and adult individuals to the more complex and heterogeneous nature of age-related conditions of advanced age. In addition, it is important to improve our understanding of measurements able to capture the conditions of interest in order to promote their optimal translation from research into clinical practice. Practical issues such as cost effectiveness also need to be considered.

Moreover, current biomarker discovery efforts have been limited by being based on predefined hypotheses. Broader screening of potential biomarkers through omics and an integrated bioinformatics approaches could advance discovery efforts. Since frailty is a failure of many systems, panels of biomarkers will likely be required. Machine learning and information technology innovation could thus be used to develop risk scores that could be used in clinical and research settings. Other technologies, such as induced pluripotent stem cells (iPSCs), could be used to study markers of senescence and could also enable a move towards personalized medicine.

*Conflicts of interest:* The Task Force was partially funded by one educational grant, Aging In Motion, and registration fees from industrial participants (Biogen, Biophytis, Cytokinetics, Glaxosmithkline, Longeveron, Pfizer and Rejuvenate Biomed NV). These corporations placed no restrictions on this work. L. Rodriguez Mañas, M. Cesari, M Pahor, J. Walston declare there are no conflicts. S. Bhasin reports grants from AbbVie, grants from Alivegen, grants from MIB, grants from Abbott, other from FPT, other from AbbVie, outside the submitted work. He has a patent Free testosterone determination issued. Y. Rolland reports grants from Biophytis, Novartis, outside the submitted work. R. Fielding reports grants from National Institutes of Health (National Institute on Aging), during the conduct of the study; grants, personal fees and other from Axcella Health, other from Inside Tracker, grants and personal fees from Biophytis, grants and personal fees from Astellas, personal fees from Cytokinetics, personal fees from Amazentis, grants and personal fees from Nestle', personal fees from Glaxo Smith Kline, outside the submitted work. B. Vellas reports grants from Nestle, Nutricia, Novartis outside the submitted work.

## References

[1] Calvani R, Marini F, Cesari M (2015). Biomarkers for physical frailty and sarcopenia: state of the science and future developments. J Cachexia Sarcopenia Muscle.

[2] Rosenberg IH (1997). Sarcopenia: origins and clinical relevance. J Nutr.

[3] Clark BC (2019). Neuromuscular Changes with Aging and Sarcopenia. J Frailty Aging.

[4] Vellas B, Pahor M, Manini T (2013). Designing pharmaceutical trials for sarcopenia in frail older adults: EU/US Task Force recommendations. J Nutr Health Aging.

[5] Woo T, Yu S, Visvanathan R (2016). Systematic Literature Review on the Relationship Between Biomarkers of Sarcopenia and Quality of Life in Older People. J Frailty Aging.

[6] Pahor M, Kritchevsky SB, Waters DL (2018). Designing Drug Trials for Frailty: ICFSR Task Force 2018. J Frailty Aging.

[7] Gobbens RJ, Luijkx KG, Wijnen-Sponselee MT, Schols JM (2010). Towards an integral conceptual model of frailty. J Nutr Health Aging.

[8] Rockwood K, Song X, MacKnight C (2005). A global clinical measure of fitness and frailty in elderly people. CMAJ.

[9] Rodriguez-Manas L, Feart C, Mann G (2013). Searching for an operational definition of frailty: a Delphi method based consensus statement: the frailty operative definition-consensus conference project. J Gerontol A Biol Sci Med Sci.

[10] Fried LP, Tangen CM, Walston J (2001). Frailty in older adults: evidence for a phenotype. J Gerontol A Biol Sci Med Sci.

[11] Cao L, Morley JE (2016). Sarcopenia Is Recognized as an Independent Condition by an International Classification of Disease, Tenth Revision, Clinical Modification (ICD-10-CM) Code. J Am Med Dir Assoc.

[12] Vellas B, Fielding RA, Bens C (2018). Implications of ICD-10 for Sarcopenia Clinical Practice and Clinical Trials: Report by the International Conference on Frailty and Sarcopenia Research Task Force. J Frailty Aging.

[13] Landi F, Calvani R, Cesari M (2015). Sarcopenia as the Biological Substrate of Physical Frailty. Clin Geriatr Med.

[14] Cesari M, Landi F, Calvani R (2017). Rationale for a preliminary operational definition of physical frailty and sarcopenia in the SPRINTT trial. Aging Clin Exp Res.

[15] Marzetti E, Calvani R, Cesari M (2015). Operationalization of the physical frailty & sarcopenia syndrome: rationale and clinical implementation. Transl Med UniSa.

[16] Marzetti E, Picca A, Marini F (2019). Inflammatory signatures in older persons with physical frailty and sarcopenia: The frailty “cytokinome” at its core. Exp Gerontol.

[17] Semba RD, Gonzalez-Freire M, Tanaka T (2019). Elevated Plasma Growth and Differentiation Factor-15 is Associated with Slower Gait Speed and Lower Physical Performance in Healthy Community-Dwelling Adults. J Gerontol A Biol Sci Med Sci.

[18] Collerton J, Martin-Ruiz C, Davies K (2012). Frailty and the role of inflammation, immunosenescence and cellular ageing in the very old: cross-sectional findings from the Newcastle 85+ Study. Mech Ageing Dev.

[19] Studenski SA, Peters KW, Alley DE (2014). The FNIH sarcopenia project: rationale, study description, conference recommendations, and final estimates. J Gerontol A Biol Sci Med Sci.

[20] Cawthon PM, Orwoll ES, Peters KE (2018). Strong Relation between Muscle Mass Determined by D3-creatine Dilution, Physical Performance and Incidence of Falls and Mobility Limitations in a Prospective Cohort of Older Men. J Gerontol A Biol Sci Med Sci.

[21] Heymsfield SB, Arteaga C, McManus C, Smith J, Moffitt S (1983). Measurement of muscle mass in humans: validity of the 24-hour urinary creatinine method. Am J Clin Nutr.

[22] Clark RV, Walker AC, O'Connor-Semmes RL (2014). Total body skeletal muscle mass: estimation by creatine (methyl-d3) dilution in humans. J Appl Physiol (1985).

[23] Evans WJ, Hellerstein M, Orwoll E, Cummings S, Cawthon PM (2019). D3 -Creatine dilution and the importance of accuracy in the assessment of skeletal muscle mass. J Cachexia Sarcopenia Muscle.

[24] Cawthon PM, Blackwell T, Cummings SR (2019). The association between muscle mass assessed by D3CR dilution with incident ADL and IADL disability in community dwelling older men. J Frailty Aging.

[25] Ko F, Abadir P, Marx R (2016). Impaired mitochondrial degradation by autophagy in the skeletal muscle of the aged female interleukin 10 null mouse. Exp Gerontol.

[26] Varadhan R, Yao W, Matteini A (2014). Simple biologically informed inflammatory index of two serum cytokines predicts 10 year all-cause mortality in older adults. J Gerontol A Biol Sci Med Sci.

[27] Hellhammer DH, Wust S, Kudielka BM (2009). Salivary cortisol as a biomarker in stress research. Psychoneuroendocrinology.

[28] Johar H, Emeny RT, Bidlingmaier M (2014). Blunted diurnal cortisol pattern is associated with frailty: a cross-sectional study of 745 participants aged 65 to 90 years. J Clin Endocrinol Metab.

[29] Varadhan R, Walston J, Cappola AR, Carlson MC, Wand GS, Fried LP (2008). Higher levels and blunted diurnal variation of cortisol in frail older women. J Gerontol A Biol Sci Med Sci.

[30] Doi T, Makizako H, Tsutsumimoto K (2018). Association between Insulin-Like Growth Factor-1 and Frailty among Older Adults. J Nutr Health Aging.

[31] Carcaillon L, Blanco C, Alonso-Bouzon C, Alfaro-Acha A, Garcia-Garcia FJ, Rodriguez-Manas L (2012). Sex differences in the association between serum levels of testosterone and frailty in an elderly population: the Toledo Study for Healthy Aging. PLoS One.

[32] Carcaillon L, Garcia-Garcia FJ, Tresguerres JA, Gutierrez Avila G, Kireev R, Rodriguez-Manas L (2012). Higher levels of endogenous estradiol are associated with frailty in postmenopausal women from the toledo study for healthy aging. J Clin Endocrinol Metab.

[33] Ma L, Niu H, Sha G, Zhang Y, Liu P, Li Y (2019). Serum SIRT1 Is Associated with Frailty and Adipokines in Older Adults. J Nutr Health Aging.

[34] Kalyani RR, Varadhan R, Weiss CO, Fried LP, Cappola AR (2012). Frailty status and altered glucose-insulin dynamics. J Gerontol A Biol Sci Med Sci.

[35] Leng SX, Cappola AR, Andersen RE (2004). Serum levels of insulin-like growth factor-I (IGF-I) and dehydroepiandrosterone sulfate (DHEA-S), and their relationships with serum interleukin-6, in the geriatric syndrome of frailty. Aging Clin Exp Res.

[36] Alonso-Bouzon C, Carcaillon L, Garcia-Garcia FJ, Amor-Andres MS, El Assar M, Rodriguez-Manas L (2014). Association between endothelial dysfunction and frailty: the Toledo Study for Healthy Aging. Age (Dordr).

[37] Walston J, McBurnie MA, Newman A (2002). Frailty and activation of the inflammation and coagulation systems with and without clinical comorbidities: results from the Cardiovascular Health Study. Arch Intern Med.

[38] Andreux PA, van Diemen MPJ, Heezen MR (2018). Mitochondrial function is impaired in the skeletal muscle of pre-frail elderly. Sci Rep.

[39] Corona G, Polesel J, Fratino L (2014). Metabolomics biomarkers of frailty in elderly breast cancer patients. J Cell Physiol.

[40] Burch JB, Augustine AD, Frieden LA (2014). Advances in geroscience: impact on healthspan and chronic disease. J Gerontol A Biol Sci Med Sci.

[41] Epel ES, Lithgow GJ (2014). Stress biology and aging mechanisms: toward understanding the deep connection between adaptation to stress and longevity. J Gerontol A Biol Sci Med Sci.

[42] Newgard CB, Pessin JE (2014). Recent progress in metabolic signaling pathways regulating aging and life span. J Gerontol A Biol Sci Med Sci.

[43] Scaffidi P, Misteli T (2006). Lamin A-dependent nuclear defects in human aging. Science.

[44] El Assar M, Angulo J, Carnicero JA (2017). Frailty Is Associated With Lower Expression of Genes Involved in Cellular Response to Stress: Results From the Toledo Study for Healthy Aging. J Am Med Dir Assoc.

[45] Ciabattini A, Nardini C, Santoro F, Garagnani P, Franceschi C, Medaglini D (2018). Vaccination in the elderly: The challenge of immune changes with aging. Semin Immunol.

[46] Lopez-Otin C, Blasco MA, Partridge L, Serrano M, Kroemer G (2013). The hallmarks of aging. Cell.

[47] Simon AK, Hollander GA, McMichael A (2015). Evolution of the immune system in humans from infancy to old age. Proc Biol Sci.

[48] Marzetti E, Calvani R, Landi F (2015). Innovative Medicines Initiative: The SPRINTT Project. J Frailty Aging.

[49] Calvani R, Picca A, Marini F (2018). The “BIOmarkers associated with Sarcopenia and PHysical frailty in EldeRly pErsons” (BIOSPHERE) study: Rationale, design and methods. Eur J Intern Med.

[50] Erusalimsky JD, Grillari J, Grune T (2016). In Search of ‘Omics'-Based Biomarkers to Predict Risk of Frailty and Its Consequences in Older Individuals: The FRAILOMIC Initiative. Gerontology.

[51] Rodriguez-Manas L (2015). Use of Biomarkers. J Frailty Aging.

